# Laying the Foundations for a Human-Predator Conflict Solution: Assessing the Impact of Bonelli's Eagle on Rabbits and Partridges

**DOI:** 10.1371/journal.pone.0022851

**Published:** 2011-07-27

**Authors:** Marcos Moleón, José A. Sánchez-Zapata, José M. Gil-Sánchez, José M. Barea-Azcón, Elena Ballesteros-Duperón, Emilio Virgós

**Affiliations:** 1 Empresa de Gestión Medioambiental-Consejería de Medio Ambiente, Junta de Andalucía, Granada, Spain; 2 Departamento de Biología Aplicada, Universidad Miguel Hernández, Orihuela, Alicante, Spain; 3 Departamento de Biología y Geología, Escuela Superior de Ciencias Experimentales y Tecnología, Universidad Rey Juan Carlos, Móstoles, Madrid, Spain; Albert Einstein College of Medicine, United States of America

## Abstract

**Background:**

Predation may potentially lead to negative effects on both prey (directly via predators) and predators (indirectly via human persecution). Predation pressure studies are, therefore, of major interest in the fields of theoretical knowledge and conservation of prey or predator species, with wide ramifications and profound implications in human-wildlife conflicts. However, detailed works on this issue in highly valuable –in conservation terms– Mediterranean ecosystems are virtually absent. This paper explores the predator-hunting conflict by examining a paradigmatic, Mediterranean-wide (endangered) predator-two prey (small game) system.

**Methodology/Principal Findings:**

We estimated the predation impact (‘kill rate’ and ‘predation rate’, i.e., number of prey and proportion of the prey population eaten, respectively) of Bonelli's eagle *Aquila fasciata* on rabbit *Oryctolagus cuniculus* and red-legged partridge *Alectoris rufa* populations in two seasons (the eagle's breeding and non-breeding periods, 100 days each) in SE Spain. The mean estimated kill rate by the seven eagle reproductive units in the study area was c. 304 rabbits and c. 262 partridges in the breeding season, and c. 237 rabbits and c. 121 partridges in the non-breeding period. This resulted in very low predation rates (range: 0.3–2.5%) for both prey and seasons.

**Conclusions/Significance:**

The potential role of Bonelli's eagles as a limiting factor for rabbits and partridges at the population scale was very poor. The conflict between game profitability and conservation interest of either prey or predators is apparently very localised, and eagles, quarry species and game interests seem compatible in most of the study area. Currently, both the persecution and negative perception of Bonelli's eagle (the ‘partridge-eating eagle’ in Spanish) have a null theoretical basis in most of this area.

## Introduction

Although predation is one of the most frequent multi-species interactions in natural systems [1], very little is still known about its ecological consequences and, more specifically, no consensus has been reached about its potential as a limiting factor. However, it seems irrefutable that, at least under certain circumstances (e.g., low prey densities and high predation rates), predators are capable of notably exerting an influence on the population dynamics of their prey species (see the reviews in [2]–[5]).

Nevertheless, predation is not only a controversial topic in theoretical fields as vertebrate predators also interact with human interests; for instance livestock rearing and hunting. Predation of game species leads to serious conservation problems in relation to numerous threatened predators as it leads to humans persistently persecuting them [6]–[8], [9], [10]. However, the persecution levels that predators have traditionally been subjected to are disproportionately excessive when considered in the light of the scarce current ecological foundation provided ([11], [12]; but see [3]).

An understanding of the limiting potential that predators exert on their prey must be supported by profound knowledge of the predation impact. This concept is understood as the amount of the prey population taken by the predator [13], [14]. However, considerable deficiencies can currently be identified in our knowledge of the predation impact. For instance, a strong geographical bias is obvious as very little information about warm ecosystems is available other than African savannahs. Besides Africa (e.g., [15]), the few studies available on this subject in the scientific literature have been almost exclusively conducted in northern latitudes of Europe and North America (e.g., [3]). Since the final result of each specific interaction depends on its ecological context, applying the conclusions drawn from these studies to other ecosystems usually proves difficult. Therefore, undertaking studies in, for example Mediterranean climates, has been considered an urgent necessity [3].

In this study we explore the predator-hunting conflict by examining a paradigmatic, Mediterranean-wide (endangered) predator-two prey system.

### The conflict

In the Iberian Peninsula, the red-legged partridge *Alectoris rufa*, and particularly the rabbit *Oryctolagus cuniculus*, are crucial prey for the large community of vertebrate predators (see the up-to-date reviews in [16], [17]). Thus the population viability of many raptors and carnivores largely depends on the management and conservation of these prey species. In parallel, rabbits and partridges are the most valuable small game species in hunting terms in the Iberian countries, with more than 4 million and 3 million, respectively, shot annually in Spain (Spanish Ministry of Environment, and Rural and Marine Affairs, http://www.marm.es/). Moreover, areas dedicated to small game hunting profusely spread throughout the Peninsula (up to 80% of the surface area in some regions; [18]), where hunting activity is a traditional yet increasingly important economic resource and pastime activity [19]. In fact, some agro-environmental schemes are highly dependent on hunting for survival, and small-game activities often require increased hunting yields to guarantee future profitability [9]. As expected, the illegal persecution of raptors and carnivores by hunters and game managers is a long-term habitual practice in this area [9], [20]. Until the late 1960s, the Spanish government paid to kill predator species, which are now threatened and even critically endangered. After banning killing schemes, further illegal hunting and poisoning of predators has continued until the present-day [21], [22]. For conservationists, such human pressure implies serious concerns as it has been shown to affect the large-scale spatio-temporal population dynamics of high conservation value species (e.g., [23]). Moreover, this is an outstanding conflict not only from a local, national perspective because a large number of Spanish predators are endangered at both the national and international levels, and Spain houses the largest European and even world populations of many of them (see Table S1).

Bonelli's eagle *Aquila fasciata* is a good example of this situation. This species, with three quarters of the European population in Spain, is a threatened raptor in Mediterranean countries, and is considered “endangered” in Europe (SPEC 3 level; [24]; Table S1). One of the most important underlying causes is non-natural mortality directly related to hunting and game management [25]. In Spain, Bonelli's eagle is the principal known predator of adult red-legged partridges and one of the most important consumers of rabbits (see the up-to-date reviews in [16], [26]). Yet despite the apparent connection between this raptor's feeding habits and a large part of the persecution it suffers, an approximation of this species's predation impact on rabbits or partridges has never been done.

Here we estimate the predation impact (the ‘kill rate’ –number of prey consumed by the predator– and the ‘predation rate’ –percentage of the prey population consumed by the predator–; e.g. [3]) of Bonelli's eagle on rabbit and partridge populations during two periods (the eagle's breeding and non-breeding seasons). This was performed in a typically Mediterranean habitat in SE Spain for the ultimate purpose of being able to contribute to the design of ecologically-based strategies that reconcile the conservation of raptors and game species with game management in such environments.

## Materials and Methods

### Ethics Statement

All the work was conducted in accordance with relevant national and international guidelines, and conforms to the legal requirements of the regional government (Regional Environmental Ministry of the Junta de Andalucía; permits A and B). Likewise, all efforts were made to minimize animals' suffering. All the work involving the manipulation of wild eagles (feeding trials) was supervised by trained veterinarians. Chicks involved in the feeding trials were taken from the wild for a reintroduction programme of the species in France (permission was obtained from the Regional Government upon the request of the Director of the Ligue pour la Protection des Oiseaux). Therefore, no additional specific approval was required by the Public Administration. In any case, optimal conditions (including food, temperature and space) were always made available to chicks, which were maintained in a centre specialising in animal care (Centro de Recuperación de Especies Amenazadas “Quiebrajano”) for the duration of the trials. Eagle nest observations were undertaken from a distance using fieldscopes (20–60×) with a view to minimising risk of disturbances. Collection of food samples in the surroundings of eagle nests was conducted once chicks had fledged.

### Study area and Bonelli's eagle population

**Figure 1:**
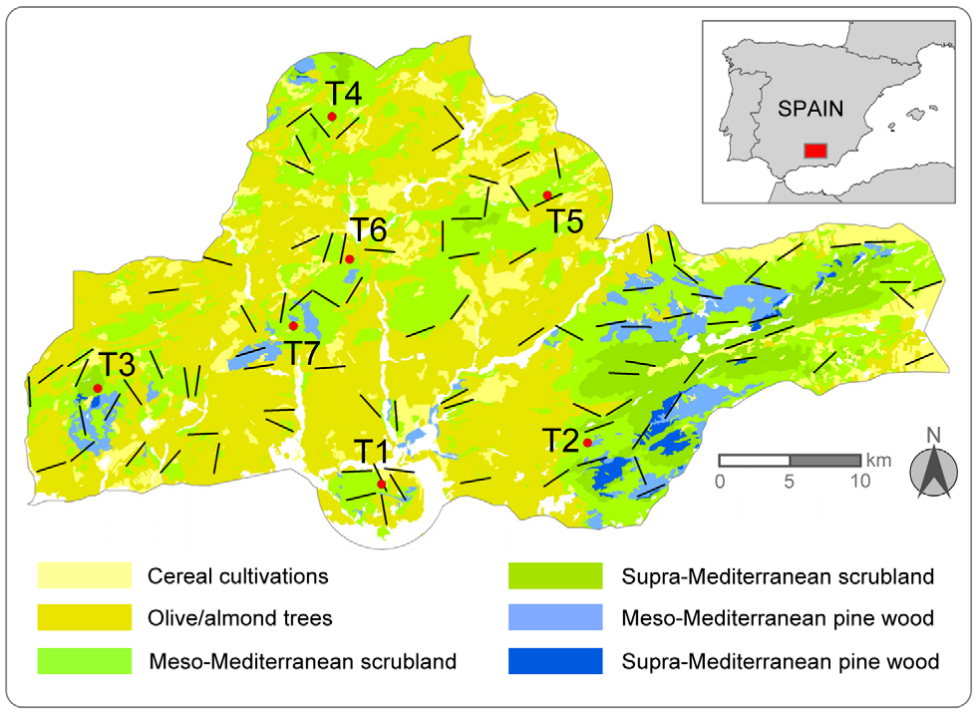
Study area. Straight black lines indicate the census transect routes. Red circles mark the nest or geometric centre of the nests used by each eagle pair during the study period. White areas indicate other habitat categories (urban or industrial areas, irrigated crops, river valleys, etc.).

The study area (121 017 ha) is located in SE Spain (Fig. 1). The altitudinal range varies between 420 and 2027 m a.s.l. A typically Mediterranean climate predominates, with average temperatures of 5.5–7.8°C in January and 25.7–26.8°C in July. Annual rainfall is 460–606 mm of irregular distribution and primarily in spring and autumn. The area is characterised by a mosaic of habitats, with stands of pines (principally *Pinus halepensis* and *P. pinaster*), patches of Mediterranean scrub with scattered holm oaks *Quercus ilex* at various grades of development, and non-irrigated arable crop lands (cereals, olives and almonds). The whole area supports intense hunting activity, mainly small game, including rabbit and partridge shooting.

The study area included seven Bonelli's eagle territories in a matrix with an unoccupied habitat. This area can be considered representative of the regional population as a whole because it holds a similar density of pairs to that for the total population in the region [27], and the habitat characteristics (vegetation, topography, degree of humanisation, etc.) are also similar to those of the rest of the population [28]. All the breeding territories were visited annually (2002–2004) to check for occupation and to register productivity (number of fledglings per pair). Since no areas for juvenile dispersion were present inside the study area [29], floating individuals were not taken into account.

### Diet and food requirements of Bonelli's eagles

Regurgitated pellet contents were used to quantify diet [30]. Following Real (1996; [30]), each prey species identified in one food pellet was counted as one individual. Regarding breeding season impact, pellets were collected in 2002, 2003 and 2004 on perches close to nests. For the impact estimate during the non-breeding period, pellets were collected in autumn (September–December) of 2003. Data were considered valid for a territory only when >20 prey were obtained (see [31] for minimum sample sizes in proportion data studies). However, the sample for the non-breeding period in 2003 was insufficient because the eagles used known perches less frequently. Consequently, the diet analysis for this period was complemented with the pellets collected in the autumns of 1998–2002 and 2004–2006. Combining these data was possible given the absence of important interannual variations in the diet of Bonelli's eagles in this population during the non-breeding period ([18]; moreover, there were no differences noted during the breeding period; [28]). Furthermore, available hunting bag statistics for the province of Granada (1998–2003) reflect strong stability in the density of the main Bonelli's eagle' prey species; i.e., rabbits and partridges (Spanish Ministry of Environment, and Rural and Marine Affairs, http://www.marm.es/).

Diet data are expressed in terms of relative frequency (%N) and percentage of biomass consumed (%B). The methodology followed to calculate the biomass consumed and eagles' food requirements is found in Text S1 and Table S2.

### Prey densities (*DP*)

Counts of rabbits and red-legged partridges were done in the springs of 2002, 2003 and 2004, and in autumn 2003. This gave a total of 99×2-km long linear transects in each period (always the same; Fig. 1). Transects were stratified by habitats [32] so that the proportion of each different habitat crossed by the transects was similar to the proportion of habitats in the study area. Paths, forest tracks, roads and level-line routes were avoided, unless a transect coincided with any of these by chance [33]. Transects were undertaken on foot by the same three observers (mean speed: 3.1 km/h; range: 1.6–4.8 km/h) in the early morning or late evening. Rainy and windy days were avoided. Prior to the census, these observers undertook five practice transects to standardise their perceptions of distances.

To calculate rabbit densities, the method of Palomares et al. (2001; [33]) was employed, an accurate method for low-medium rabbit density areas like ours in which the confident use of other modelling procedures is prevented. In agreement with these authors, rabbit abundance was estimated by the following regression line (P<0.0001, r^2^ = 0.97): “absolute rabbit density = 0.57×number of rabbits observed within 10 m of each side of the transect line per km walked”. The figure of 10 m to diminish among-habitat differences in visibility was established [33].

Partridge densities were estimated using the DISTANCE 5.0 free software [34]. Before model fitting, data were grouped into 10 m intervals to improve the model fit and to reduce measurement biases. In a first step of the analysis, five *a priori* useful models were employed: a uniform key function with either cosine or polynomial series expansion, a half-normal key function with either cosine or Hermite polynomial series expansion, and a Hazard rate key function with cosine series expansion. The detection function was obtained using all the survey transects to obtain a sufficient number of contacts in order to produce accurate estimates of population density. Model selection was based on a minimum AIC score and on chisquared goodness-of-fit tests.

In spring, censuses were undertaken between mid-March and the end of April (halfway through the eagle breeding cycle), between the end of the partridge hunting period and the start of the partridge breeding period, such that this prey's minimum annual population was estimated (the breeding stock; [35]). Given the species life cycle [35], [36], little change in density could then be estimated throughout the spring study period. For rabbits, the spring census dates coincided with the species' principal phase of annual population growth (maximum population levels are reached in June–July; [36]–[38]). In autumn, censuses were undertaken between mid-September and the end of October, at the beginning of the eagle's non-reproductive period, and coincided with both minimum rabbit population densities (see earlier references) and a phase of high partridge population levels [36].

### Predation impact and sensitivity analysis

In order to estimate the predation impact of all the reproducing pairs of Bonelli's eagle (adult pairs plus chicks in the breeding season and only adults in the non-breeding season) on the respective rabbit and partridge populations in the study area, an adaptation of the formula employed by Lindén and Wikman (1983; [39]) was used:
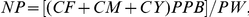
where *NP* is the number of prey (rabbits or partridges) captured by eagles, *CF* is consumption by females (number of females×length of study period×female daily dietary requirements), *CM* is consumption by males (number of males×length of study period×male daily dietary requirements), *CY* is consumption by chicks (number of chicks×consumption of a chick; calculated exclusively for the breeding period), *PPB* is proportion of the prey biomass (rabbit or partridge) in the eagle's total diet (in the breeding or the non-breeding period, depending on the case), and *PW* is the corrected average prey (rabbit or partridge) weight. *PW* represents the mean useable biomass of rabbits or partridges, i.e., 774.7 / 816.9 g and 364.8 g, respectively (see Text S1).

The predation impact was estimated for two different periods depending on eagle phenology: the breeding (in 2003, 2003 and 2004) and the non-breeding season (in 2003). The Bonelli's eagle reproductive period in the study area lasts approximately 100 days (from mid-February to the end of May), including 40 incubation days and 60 days during which chicks remain in the nest [40]. In order to make the results comparable, a 100-day period for the non-breeding period was also considered, from the beginning of September (when the juvenile dependency period had ended and the young had left the study area; [36]; pers. obs.) until mid-December (when the majority of eagles had commenced a new breeding cycle; e.g., nest construction and pair bonding; pers. obs.). This non-breeding period well overlaps the main hunting period of rabbits and partridges in S. Spain. The periods between the breeding and non-breeding seasons defined above were not included as they were expected to support intermediate predation pressure values given both the eagles' energy demand throughout the year and prey life cycles (see above).

After subjecting the estimated parameters to a sensitivity analysis (see Text S2 and Table S3), the impact results are offered as the number of rabbits / partridges consumed and the percentage of the rabbit / partridge population consumed (‘kill rate’ and ‘predation rate’, respectively) by eagles for each period (reproductive and non-reproductive).

## Results

### Bonelli's eagle population and diet

The Bonelli's eagle population in the study area remained constant in seven territories. All the pairs comprised adults. Mean productivity was 1.43 fledglings/pair in all three study years.

**Figure 2:**
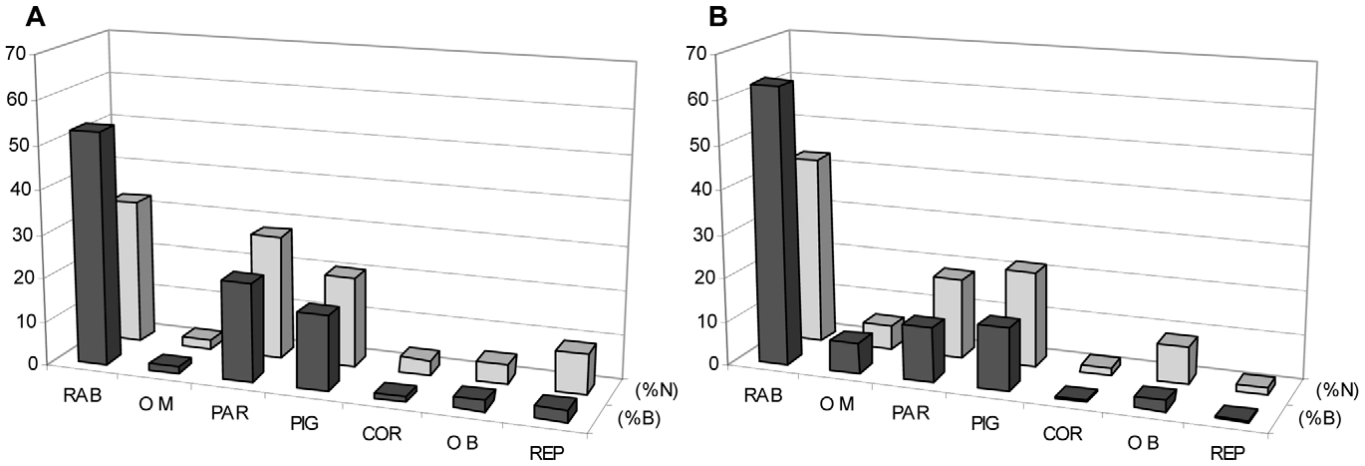
Bonelli's eagle diet in the study area during the breeding (a) and non-breeding (b) periods. Dark bars: percentage of biomass consumed (%B); light bars: relative frequency (%N); RAB: rabbit; OM: other mammals; PAR: red-legged partridge; PIG: pigeons; COR: corvids; OB: other birds; REP: reptiles.

Overall, rabbits were the principal prey in spring (n = 466 prey items; n = 6 studied pairs) in both relative frequency (32.8%) and ingested biomass (52.9%) terms. Red-legged partridges were the second most important prey during this period, with 28.0% relative frequency and 22.4% of ingested biomass terms. Pigeons (c. 70% *Columba livia* and c. 30% *C. palumbus*) were the third most frequently consumed prey, with values close to those of partridges (19.6%N and 17.1%B). The remaining prey appeared in proportions of <10%. The autumn diet (*n* = 147 prey; n = 3 studied pairs) included increased rabbit consumption (42.7%N and 63.0%B), while that of partridge decreased and dropped to become third in importance (18.3%N and 12.4%B) and below that of pigeons (21.6%N and 14.4%B). Once again, the remaining prey appeared as a low proportion (Fig. 2).

### Predation impact

The eagle population (7 pairs) in the study area consumed an average estimate of c. 304 rabbits (minimum and maximum in the three study years: c. 278–341) and c. 262 partridges (minimum and maximum in the three study years: c. 224–304) during the reproductive period, as well as c. 237 rabbits and c. 121 partridges in the non-reproductive period (Table 1). Accordingly, the total eagle population consumed an average of 1.38% of the rabbits (range at a 10% error in *DP*: 0.88–2.04%; range at a 50% error in *DP*: 0.64–2.51%) and 1.26% of the partridges (range at a 10% error in *DP*: 0.85–2.01%; range at a 50% error in *DP*: 0.62–2.47%; Table 2) censussed in the study area during the breeding period. Predation rates in the non-breeding period were 1.55% for rabbits (range at a 10% error in *DP*: 1.42–1.68%; range at a 50% error in *DP*: 1.03–2.07%) and 0.42% for partridges (range at a 10% error in *DP*: 0.38–0.46%; range at a 50% error in *DP*: 0.28–0.56%; Table 2).

**Table 1:** Frequency (relative, N, and of the biomass consumed, B) of rabbits and red-legged partridges in Bonelli's eagle diet and the kill rates of these prey in the study area.

Period	Year	No. of prey	% of rabbit in diet (N)	% of rabbit in diet (B)	No. of rabbits eaten	% of partridge in diet (N)	% of partridge in diet (B)	No. of partridges eaten
Breeding	2002	169	33.55	56.85	340.5	21.47	17.62	224.1
Breeding	2003	193	25.91	46.44	278.1	23.34	20.23	257.3
Breeding	2004	104	28.31	48.96	293.2	27.83	23.90	304.0
Non-breeding	2003	423	35.34	56.07	237.4	17.92	12.74	120.8

**Table 2:** Census results and predation rates on rabbits and red-legged partridges in the study area.

Prey	Period	Year	Km of transects	Prey density (ind./ha)	95% CI of prey density	Total no. of prey	% of the prey population eaten	% minimum and maximum predation rates at a 10% error*	% minimum and maximum predation rates at a 50% error*
Rabbit	Breeding	2002	198	0.1497	0.0879–0.2115	18116	1.88	1.72–2.04	1.25–2.51
Rabbit	Breeding	2003	198	0.1785	0.0821–0.2749	21600	1.29	1.18–1.40	0.86–1.72
Rabbit	Breeding	2004	198	0.2533	0.1379–0.3687	30658	0.96	0.88–1.04	0.64–1.28
Rabbit	Non-breeding	2003	198	0.1267	0.0233–0.2301	15329	1.55	1.42–1.68	1.03–2.07
Red-legged partridge	Breeding	2002	198	0.1879	0.1354–0.2607	22738	0.99	0.91–1.07	0.66–1.32
Red-legged partridge	Breeding	2003	198	0.2291	0.0759–0.6917	27730	0.93	0.85–1.01	0.62–1.24
Red-legged partridge	Breeding	2004	198	0.1357	0.0384–0.4798	16421	1.85	1.69–2.01	1.23–2.47
Red-legged partridge	Non-breeding	2003	198	0.2356	0.1473–0.3454	28506	0.42	0.38–0.46	0.28–0.56

* The minimum and maximum values in the predation rates were obtained by applying errors of 10% and 50% to the mean rabbit/partridge density estimated in the study area (see Text S2 for further details).

## Discussion

Bonelli's eagle predation rates on rabbits and red-legged partridges in the study area were extremely low (0.3–2.5%; see Text S3 for potential biases in estimates) for both the breeding and non-breeding periods. For partridges, these figures were generally well below those reported by other studies of predator impact by raptors on game birds in Europe (Table S4), and were at least one order lower in magnitude than those mentioned in studies showing consistent evidence for prey limitation ([14], [41]; range of spring predation rates on adult birds for these studies: 11–32%). Bonelli's eagle's low population density deriving from its greater territorial requirements compared to other raptors, can be considered the principal cause of its low predation impact on partridges. For example, goshawk density in Finland was almost 10 times higher than that of Bonelli's eagles in our study area [39], and those of falcons and harriers in Scotland were 5–17 and 10–36 times higher, respectively [42]. There are no specific European studies available on the impact of raptors on rabbits, and the very few studies which deal with the effect of all predators (mammals and raptors) on this species in Europe (conducted outside the Mediterranean range) indicate that predation has a minor influence, or may be even limiting but only at very low rabbit densities [43], [44].

If we remove the effect of nestling prey intake, the rabbit kill rates of Bonelli's eagles were practically the same between the non-breeding period studied and both the previous and subsequent breeding periods. The predation rate values obtained were distinct given seasonal differences in the rabbit population. Thus, the impact of adult eagles was higher during the non-breeding season than during both the preceding and following breeding seasons. The increase noted in rabbits' relative consumption in autumn, when rabbits are scarcer, may be in agreement with the hypothesis provided in earlier works, indicating Bonelli's eagle's preference to this prey [26], [45]–[47]. Nonetheless, the lesser vulnerability of partridge males during this season (see below) could also contribute [46], [47].

In contrast to rabbits, the partridge kill and predation rates were higher in spring than in autumn. During the eagle's breeding season, this predation was biased towards males (see Text S1), as shown in other areas of the Iberian Peninsula [48]. Male partridges' conspicuous behaviour during the breeding season, a period when they spend considerable time singing on unprotected, highly visible perches, could make them especially vulnerable to predators [49]. Therefore, this hypothesis could be a reason for the higher pressure exerted on partridges by Bonelli's eagles in spring.

The differential predation of sexes should be added to the final predation effect on the partridge population as it may act by lowering the partridge population growth rate through an unpaired sex ratio. Yet it seems unlikely that the elimination of males could reach high enough levels to significantly reduce the reproductive potential of the whole partridge population at the very low mean predation levels presented herein. In addition females of ground-nesting birds, such as partridges, are more likely to be captured by terrestrial predators during the breeding period than males [50], [51]; this situation could partially compensate the bias induced by avian predators like the Bonelli's eagle.

### Synthesis and conservation implications

Here, for the first time, we present a key piece of the predator-hunting conflict puzzle in Spain after having examined a predator of high conservation value on the continental scale and the two most valuable local small-game prey species. The capacity of Bonelli's eagle to limit rabbits and partridges in the study area can be considered as very poor at the population level. Furthermore, hunting estates with strong hunting pressure that obtain more economic benefits are typically those with higher game species densities ([9]; pers. obs.). Therefore, in those areas where conflict is potentially more plausible, the predation pressure by Bonelli's eagles would actually be the lowest. Besides, the average size of hunting estates in the study area is 850 ha; thus, the occurrence of a kill rate extreme (overall 49 rabbits and 40 partridges in a 100-day period per eagle pair plus chicks) on a single estate would be most unlikely or at least extremely rare, given the much larger territory normally used by eagles (median home range: 3,610–5,030 ha; [52]). This implies that the vast majority of the hunting estates located in the study area would be free from ‘competition’ by Bonelli's eagle, leading to a significant reduction in the conflicting interests of hunting and conservation lobbies. Consequently, the persecution and the negative perception of Bonelli's eagle currently have a null theoretical basis in most of the study area.

The impact of Bonelli's eagle on the autumn-winter hunting bag of red-legged partridge is likely to be fairly lower than that estimated for other predators feeding on game birds (e.g. [3]) given the notably lower impact on the breeding stock (and the resulting chick production) in preceding springs. Moreover, the estimated impact of eagles coinciding with the main small-game hunting period in the study area shows even lower predation pressure on partridges than in spring. In addition, partridge hunting using call lures (January–March; a common, economically important practice in S. Spain consisting in hunters hidden in front of a singing captive male partridge, which attracts wild males for shooting) would be even less affected by eagles, as only adult partridges are subject to this hunting form (i.e., no stock of potential chicks should be added to the predation levels estimated for this period; moreover, the breeding birds' density in a given year is much less dependent than the autumn one on productivity in the previous spring; e.g., [3]). This last finding is particularly relevant for Bonelli's eagle conservation since it has been suggested that this deeply-rooted hunting technique could cause the loss of an considerable number of breeding eagles (hunters shoot eagles while waiting for partridges) and of the subsequent disappearance of some breeding territories [53]. In general, the rabbit hunting bag would be scarcely affected given this prey's breeding potential and the low values found in the number of individuals taken from the huntable stock ([37]; see before and Text S3 for further discussion).

It is worth highlighting that the Bonelli's eagle population density in our study area is one of the highest in Europe, and that its productivity is the highest ever recorded for this species [27], [28]. Therefore, the impact in other areas with similar or greater densities of rabbits and partridges (e.g., in the majority of the C and S of the Iberian Peninsula; [54], [55]) is expected to be even lower.

Regarding the possibility of extrapolating our results to other Mediterranean predators, in the specific case of the red-legged partridge, Bonelli's eagle is the Iberian predator whose diet markedly includes more adults as prey [16]. Consequently, only those partridge predators reaching much higher densities than Bonelli's eagles (only the eagle owl *Bubo bubo* in our study area; [53]) would be able to exert greater pressure on adult partridges, which are precisely those with a greater reproductive potential in the population. Our results also suggest that large raptors such as eagles, precisely the most persecuted species, would have a reduced global impact on game populations if compared to species with smaller territories. Considering, moreover, that large raptors usually kill smaller predators (e.g., [26], [56]–[58]), there seem to be multiple reasons to conserve healthy populations of large eagles in hunting exploitation areas.

The fact that the partridge is not the Bonelli's eagle's principal prey [26], [46], [47], in combination with this raptor's low potential impact on this species (this study), suggests that the vernacular name of the eagle in Spanish (literally, the ‘partridge-eating eagle’) could actually overestimate its role as a limiting factor in the partridge population dynamics; thus, such an apparently trivial factor, its common name, may have traditionally favoured its persecution.

## Supporting Information

Table S1 **Spanish predators including >5% of rabbit (“rabbit consumers”) and red-legged partridge (“partridge consumers”) in their diet, together with their national and international conservation statuses.**
(DOC)

Table S2 **Prey weighed to calculate the biomass ingested by eagles.**
(DOC)

Table S3 **Results of the sensitivity evaluation of each parameter in the Lindén & Wikman (1983) equation.**
(DOC)

Table S4 **Raptor predation rates on wild populations of adult game birds in Europe.**
(DOC)

Text S1 **Calculation of the biomass consumed and food requirements of Bonelli's eagles.**
(DOC)

Text S2 **Sensitivity analysis of the impact estimates.**
(DOC)

Text S3 **Biases in the impact estimates.**
(DOC)
