# Supplementary material for: Laying the Foundations for a Human-Predator Conflict Solution: Assessing the Impact of Bonelli's Eagle on Rabbits and Partridges
Source: PLoS One. 2011 Jul 27;6(7):e22851. doi: 10.1371/journal.pone.0022851 (PMC3144957; doi:10.1371/journal.pone.0022851)
Supplement: Table S1 — Spanish predators including >5% of rabbit (“rabbit consumers”) and red-legged partridge (“partridge consumers”) in their diet, together with their national and international conservation statuses. (DOC) [file pone.0022851.s001.doc]

**Table S1.** Spanish predators including >5% of rabbit (“rabbit consumers”) and red-legged partridge (“partridge consumers”) in their diet in at least one site or season (n = 23 spp; taken from [1]), together with their national and international conservation statuses.

| **Species** | **Rabbit consumer** | **Partridge consumer** | **European population in Spain (for raptors: [2,3]; for carnivores: [4])** | **Spanish IUCN conservation status (for raptors: [3]; for carnivores: [4])** | **European IUCN conservation status (for raptors: [5]; for carnivores: [6])** | **European conservation status [5]** | **World IUCN conservation status [7]** |
| --- | --- | --- | --- | --- | --- | --- | --- |
| *Milvus migrans* | Yes | - | 7% | NT | VU | SPEC 3 (VU) | LC |
| *M. milvus* | Yes | - | 10% | EN | LC | SPEC 2 (DE) | NT |
| *Neophron percnopterus* | Yes | - | 46% | EN | EN | SPEC 3 (EN) | EN |
| *Aegypius monachus* | Yes | - | 82% | VU | LC | SPEC 1 (RA) | NT |
| *Circus aeruginosus* | Yes | Yes | 2% | NE | LC | non-SPEC | LC |
| *Accipiter gentilis* | Yes | Yes | 3% | LC | LC | non-SPEC | LC |
| *Buteo buteo* | Yes | Yes | 2% | LC | LC | non-SPEC | LC |
| *Aquila adalberti* | Yes | - | 99%^1^ | EN | EN | SPEC 1 (EN) | VU |
| *A. chrysaetos* | Yes | Yes | 19% | NT | LC | SPEC 3 (RA) | LC |
| *Hieraaetus pennatus* | Yes | Yes | 81% | LC | LC | SPEC 3 (RA) | LC |
| *H. fasciatus* | Yes | Yes | 76% | EN | EN | SPEC 3 (EN) | LC |
| *Bubo bubo* | Yes | - | 20% | LC | LC | SPEC 3 (DEP) | LC |
| *Strix aluco* | Yes | - | 5% | LC | LC | non-SPEC^E^ (SE) | LC |
| *Canis lupus* | Yes | - | ¿? | NT | LC | - | LC |
| *Vulpes vulpes* | Yes | - | ¿? | LC | LC | - | LC |
| *Mustela nivalis* | Yes | Yes | ¿? | LC | LC | - | LC |
| *M. putorius* | Yes | Yes | ¿? | NT | LC | - | LC |
| *Martes foina* | Yes | - | ¿? | LC | LC | - | LC |
| *Meles meles* | Yes | - | ¿? | LC | LC | - | LC |
| *Herpestes ichneumon* | Yes | - | most^2^ | LC | LC | - | LC |
| *Genetta genetta* | Yes | - | most^3^ | LC | LC | - | LC |
| *Felis silvestris* | Yes | - | ¿? | NT | LC | - | LC |
| *Lynx pardinus* | Yes | Yes | 100%^4^ | CR | CR | - | CR |

Egg predation was not considered. The proportion of the European population of each species living in Spain as well as the species conservation status at national (Spain), continental (Europe) and world levels are shown. CR: critically endangered; EN: endangered; VU: vulnerable; NT: near threatened; LC: least concern; NE: not evaluated; SPEC 1: European species of global conservation concern; SPEC 2: unfavourable conservation status in Europe, concentrated in Europe; SPEC 3: unfavourable conservation status in Europe, not concentrated in Europe; non-SPEC^E^: favourable conservation status in Europe, concentrated in Europe; non-SPEC: favourable conservation status in Europe, not concentrated in Europe; RA: rare; DE: declining; DEP: depleted; SE: secure.

^1^ Iberian endemism

^2^ Restricted to the Iberian Peninsula (Spain and Portugal)

^3^ Restricted to the Iberian Peninsula (Spain and Portugal) and France

^4^ Iberian endemism currently restricted to Spain

**References**

1. Moleón M, Almaraz P, Sánchez-Zapata JA (2008) An emerging infectious disease triggering large-scale hyperpredation. PLoS ONE 3: e2307.
2. Martí R, del Moral JC, eds (2003) Atlas de las Aves Reproductoras de España. Madrid: DGCN-SEO/Birdlife. 733 p.
3. Madroño A, González C, Atienza JC, eds (2004) Libro Rojo de las Aves de España. Madrid: DGB-SEO/Birdlife. 452 p.
4. Palomo LJ, Gisbert J, Blanco JC (2007) Atlas y Libro Rojo de los Mamíferos Terrestres de España. Madrid: DGB-SECEM-SECEMU. 586 p.
5. BirdLife International (2004) Birds in Europe. Population estimates, trends and conservation status, BirdLife Conservation Series No. 12. Cambridge: BirdLife International. 374 p.
6. Temple HJ, Terry A, compilers (2007) The Status and Distribution of European Mammals. Luxembourg: IUCN-Office for Official Publications of the European Communities. 45 p.
7. IUCN (2009) IUCN Red List of Threatened Species. Version 2009.2. Available: http://[www.iucnredlist.org](http://www.iucnredlist.org/) via the Internet. Accessed: 2 July 2009.
