# Supplementary material for: Laying the Foundations for a Human-Predator Conflict Solution: Assessing the Impact of Bonelli's Eagle on Rabbits and Partridges
Source: PLoS One. 2011 Jul 27;6(7):e22851. doi: 10.1371/journal.pone.0022851 (PMC3144957; doi:10.1371/journal.pone.0022851)
Supplement: Table S2 — Prey weighed to calculate the biomass ingested by eagles. (DOC) [file pone.0022851.s002.doc]

**Table S2.** Prey weighed to calculate the biomass ingested by eagles.

|  | **Total weight (g)** | **Corrected weight (g)** |
| --- | --- | --- |
| *Alectoris rufa* 1 (♀) | 344 | 290 |
| *Alectoris rufa* 2 (♀) | 427 | 366 |
| *Alectoris rufa* 3 (♀) | 387 | 331 |
| *Alectoris rufa* 4 (♀) | 409 | 347 |
| *Alectoris rufa* 5 (♀) | 374 | 332 |
| *Alectoris rufa* 6 (♂) | 426 | 359 |
| *Alectoris rufa* 7 (♂) | 451 | 369 |
| *Alectoris rufa* 8 (♂) | 488 | 413 |
| *Columba palumbus* 1 | 409 | 340 |
| *Columba palumbus* 2 | 428 | 349 |
| *Columba palumbus* 3 | 428 | 344 |
| *Columba palumbus* 4 | 408 | 322 |
| *Streptopelia turtur* 1 | 132 | 98 |
| *Streptopelia turtur* 2 | 160 | 128 |
| *Streptopelia turtur* 3 | 165 | 126 |

These were recorded using an electronic precision balance (precision: ±0.5 g). The corrected weight refers to the individual without the parts normally rejected by the eagles, i.e. large feathers (primaries and rectrices), legs, keel and viscera.
