# Supplementary material for: Laying the Foundations for a Human-Predator Conflict Solution: Assessing the Impact of Bonelli's Eagle on Rabbits and Partridges
Source: PLoS One. 2011 Jul 27;6(7):e22851. doi: 10.1371/journal.pone.0022851 (PMC3144957; doi:10.1371/journal.pone.0022851)
Supplement: Table S3 — Results of the sensitivity evaluation of each parameter in the Lindén & Wikman (1983) equation. (DOC) [file pone.0022851.s003.doc]

**Table S3.** Results of the sensitivity evaluation of each parameter in the Lindén and Wikman (1983; [1]) equation.

| **Parameter** | **Error in the parameter** | **Resulting error in the kill rate** | **Resulting error in the predation rate** |
| --- | --- | --- | --- |
| *CF/CM* | 1 | 0.37 | 0.18 |
| *CY* | 1 | 0.26 | 0.06 |
| *PPB* | 1 | 1.00 | 0.81 |
| *PW* | 1 | 0.99 | 1.18 |
| *DP* | 1 |  | 1.18 |
| *CF/CM* | 5 | 1.86 | 1.67 |
| *CY* | 5 | 1.27 | 1.08 |
| *PPB* | 5 | 5.00 | 4.80 |
| *PW* | 5 | 4.76 | 4.94 |
| *DP* | 5 |  | 4.94 |
| *CF/CM* | 10 | 3.73 | 3.88 |
| *CY* | 10 | 2.55 | 2.33 |
| *PPB* | 10 | 10.00 | 9.88 |
| *PW* | 10 | 9.09 | 9.30 |
| *DP* | 10 |  | 8.53 |
| *CF/CM* | 20 | 7.45 | 7.25 |
| *CY* | 20 | 5.09 | 4.90 |
| *PPB* | 20 | 20.00 | 19.77 |
| *PW* | 20 | 16.67 | 16.82 |
| *DP* | 20 |  | 16.83 |
| *CF/CM* | 50 | 18.63 | 18.41 |
| *CY* | 50 | 12.73 | 12.52 |
| *PPB* | 50 | 50.00 | 49.72 |
| *PW* | 50 | 33.33 | 33.46 |
| *DP* | 50 |  | 33.46 |

For each error given to a parameter, the resulting error in the kill and predation rates is shown. All values are percentages. *CF*: consumption by Bonelli’s eagle females; *CM*: consumption by Bonelli’s eagle males; *CY*: consumption by Bonelli’s eagle chicks; *PPB*: proportion of the prey biomass in the eagle’s diet; *PW*: corrected prey weight; *DP*: prey density (see text for further details).

**References**

1. Lindén H, Wikman M (1983) Goshawk predation on tetraonids: availability of prey and diet of the predator in breeding season. J Anim Ecol 52: 953–968.
