# Supplementary material for: Laying the Foundations for a Human-Predator Conflict Solution: Assessing the Impact of Bonelli's Eagle on Rabbits and Partridges
Source: PLoS One. 2011 Jul 27;6(7):e22851. doi: 10.1371/journal.pone.0022851 (PMC3144957; doi:10.1371/journal.pone.0022851)
Supplement: Table S4 — Raptor predation rates on wild populations of adult game birds in Europe. (DOC) [file pone.0022851.s004.doc]

**Table S4.** Raptor predation rates on wild populations of adult game birds in Europe.

| **Predator** | **Prey** | **Period** | **Predation rate** | **Location** | **Reference** |
| --- | --- | --- | --- | --- | --- |
| Peregrine + hen harrier | Red grouse | Summer | 23% | Scotland | [1] |
| Peregrine + hen harrier | Red grouse | Winter | 27% | Scotland | [1] |
| Gyrfalcon | Ptarmigan | Summer | 11-32% | Iceland | [2] |
| Hen harrier | Red grouse | Summer | 7% | Scotland | [3] |
| Hen harrier + other raptors | Grey partridge (♀) | Spring + summer | 5-46% | France | [4] |
| Goshawk | Red-legged partridge | Autumn | 6% | Spain | [5] |
| Goshawk | Black grouse (♀) | Spring / summer | 25% | Sweden | [6] |
| Goshawk | Black grouse (♂) | Spring / summer | 14% | Sweden | [6] |
| Goshawk | Capercaillie (♀) | Complete year | 50% | Norway | [7] |
| Goshawk | Hazel grouse | Summer | 12% | Finland | [8] |
| Goshawk | Red grouse | Summer | 20-32% | Finland | [9] |
| Goshawk | Black grouse (♀) | Summer | 17% | Finland | [9] |
| Goshawk | Black grouse (♂) | Summer | 9% | Finland | [9] |
| Goshawk | Capercaillie (♀) | Summer | 7% | Finland | [9] |
| Common buzzard | Black grouse | Summer | 1% | Finland | [10] |
| Common buzzard | Red grouse | Summer | 1% | Finland | [10] |

**References**

1. Redpath SM, Thirgood SJ (1999) Numerical and functional responses in generalist predators: hen harriers and peregrines on Scottish grouse moors. J Anim Ecol 68: 879–892.
2. Nielsen OK (1999) Gyrfalcon predation on ptarmigan: numerical and functional response. J Anim Ecol 68: 1034–1050.
3. Picozzi N (1978) Dispersion, breeding and prey of the hen harrier (*Circus cyaneus*) in Glen Dye, Kincardineshire. Ibis 120: 489–509.
4. Bro E, Reitz F, Clobert J, Migot P, Massot M (2001) Diagnosing the environmental causes of the decline in Grey Partridge *Perdix perdix* survival in France. Ibis 143: 120–132.
5. Mañosa S (1991) Biología tròfica, ús de l´hàbitat i biología de la reproducció de l´astor *Accipiter gentilis* (Linnaeus, 1758) a la Segarra [PhD thesis]. Barcelona: University of Barcelona.
6. Widén P, Andrén P, Angelstam P, Lindtröm E (1987) The effect of prey vulnerability: goshawk predation and populations of small game. Oikos 49: 233–235.
7. Wegge P (1984) Naturlig dödelighet hos storfugl giennom aret pa Östlandet. In: Myrberget S, ed. Skogsfuglprojektet 1980-1984. Viltrapport 36. pp. 76–80.
8. Lindén H, Wikman M (1983) Goshawk predation on tetraonids: availability of prey and diet of the predator in breeding season. J Anim Ecol 52: 953–968.
9. Tornberg R (2001) Pattern of goshawk *Accipiter gentilis* predation on four forest grouse species in northern Finland. Wildlife Biol 7: 245–256.
10. Valkama J, Korpimäki E, Arroyo B, Beja P, Bretagnolle V et al. (2005) Birds of prey as limiting factors of gamebird populations in Europe: a review. Biol Rev 80: 171–203.
