# Supplementary material for: Laying the Foundations for a Human-Predator Conflict Solution: Assessing the Impact of Bonelli's Eagle on Rabbits and Partridges
Source: PLoS One. 2011 Jul 27;6(7):e22851. doi: 10.1371/journal.pone.0022851 (PMC3144957; doi:10.1371/journal.pone.0022851)
Supplement: Text S1 — Calculation of the biomass consumed and food requirements of Bonelli's eagles. (DOC) [file pone.0022851.s005.doc]

**Text S1.** Calculation of the biomass consumed and food requirements of Bonelli’s eagles.

To estimate biomass, prey weights were taken from the specimens in the collection of the Departamento de Biología Animal of the Universidad de Granada (DBAG), from published information [1] and from our own data (see Table S2). In the case of the larger species, those body parts not usually ingested by the eagles were not included in the calculation of the biomass (feathers and large bones, legs and viscera). Three age classes were considered for rabbits: juveniles (mean weight: 250 g), subadults (750 g) and adults (1250 g; [2,3]). Juvenile individuals were normally eaten completely by the eagles, while for subadults and adults, 15% of the average weight was deducted in relation to the biomass not ingested, such that the final weights used for these two age classes were 637.5 g and 1062.5 g respectively. With the objective of investigating the contribution of the different rabbit age classes to the diet of the Bonelli’s eagle, the content of the pellets was analysed as well as a study of the other prey remains also found at the perch sites. These samples were collected between 1993 and 2006 (breeding period) and 1998-2006 (non-breeding period). Specifically, the skulls, mandibles, teeth, feet and claws of all rabbits appearing in pellets or remains were examined. Given that it is likely that the pellet contents and prey remains in the field will tend to lead to under- and over- estimation, respectively, of the number of large rabbits (see above), both data sources were pooled to obtain a more realistic age ratio [4]. A total of 264 rabbits which had been eaten by eagles in the breeding season were classified to age, 26.9% of which were juveniles, 16.3% were subadults and 56.8% adults; in the non-breeding period 25% were juveniles, 10% subadults and 65% adults (n = 40). Consequently, the mean weight of each rabbit consumed by Bonelli’s eagles was 774.7 g during the breeding season and 816.9 g in the non-breeding period. The tarsi of red-legged partridge appearing in pellets and other prey remains were used to determine the contribution of each sex of this prey in the eagle’s diet [5,6]. In this way, 92 partridges consumed in the breeding period were sexed, of which 69.6% were males and 30.4% females. Using this sex ratio and the mean weights of the different partridge sexes (see Table S2), the mean weight of partridges consumed by eagles in the breeding season was 364.8 g. Given the absence of data for the non-breeding period, the same value was applied for both periods. The age was not considered for this prey species given that the majority of the partridges identified were adults or near-adults, and the weight of juvenile partridges in September-December is practically the same as for adults (S. Cabezas-Díaz and E. Virgós unpublished data).

A food intake of 247 g/day was assumed (datum from a trial of a female maintained in captivity for 30 complete and consecutive days; J. Real unpublished data) for the dietary needs of an adult female Bonelli’s eagle in the wild. Given the absence of data for the dietary needs of males, we took the same figure as for females. Although the information coming from birds maintained in captivity may lead to biases, which could underestimate (e.g. due to less physical exercise) or overestimate (e.g. due to greater stress) the consumption of birds in the wild (e.g. [7]), the estimates used here are similar to those found by Real (1987; [1]) in a study of the requirements of three Bonelli’s eagles during three days in the wild (250 g/day); Cheylan (1972; [8]) also estimated the same value for individuals of this species in the wild.

To establish the intake of Bonelli’s eagle chicks, we developed a dietary trial using seven birds in captivity (three females and four males), born in liberty (see [9] to consult details about the objectives and methodology of the captures). Given the diverse methodological restrictions [9], the chick ages ranged between 30-60 days old. During the experiment, all the chicks were fed ad libitum with domestic rabbits, and the average material ingested per chick was 262.9 g/day. No differences in intake were found between sexes (means of 273.3 g for females and 252.1 g for males) nor over the period of the trial (means of 263.9 g for chicks between 30-45 days old and 261.5 g between 45-60 days old). For the first 30 days a linear relationship was assumed between chick age and dietary requirements (see [10-12]). Consequently the average intake of a chick between 0-30 days old was 131.3 g/day. Thus, the total intake of a chick during its 60 days in the nest in S Spain [13] was 11 814 g (131.3 g x 30 days + 262.7 g x 30 days).

**References**

1. Real J (1987) Evolución cronológica del regimen alimenticio de una población de *Hieraaetus fasciatus* en Catalunya: factores causantes, adaptación y efectos. Rapaci Mediterranei 12: 185–205.
2. Donázar JA (1989) Variaciones geográficas y estacionales en la alimentación del búho real (*Bubo bubo*) en Navarra. Ardeola 36: 25–39.
3. Gil-Sánchez JM, Valenzuela G, Sánchez JF (1999) Iberian wild cat *Felis silvestris tartessia* predation on rabbit *Oryctolagus cuniculus*: functional response and age selection. Acta Theriol 44: 421–428.
4. Oro D, Tella JL (1995) A comparison of two methods to study the diet of the peregrine falcon *Falco peregrinus*. J Raptor Res 29: 207–210.
5. Donázar JA, Castién E (1989) Predación del búho real (*Bubo bubo*) sobre la perdiz roja (*Alectoris rufa*): selección de edad y sexo. Doñana, Acta Vertebrata 16: 219–225.
6. Pépin D (1985) Morphological characteristics and sex classification of red-legged partridge. J Wildlife Manage 49: 228–237.
7. Stalmaster MV, Gessaman J (1982) Food consumption and energy requirements of captive bald eagles. J Wildlife Manage 46: 646–654.
8. Cheylan G (1972) Le cycle annuel d´un couple d´aigles de Bonelli *Hieraaetus fasciatus* (Vieillot). Alauda 3: 214–234.
9. Consejería de Medio Ambiente (2006) Programa de Actuaciones para la Conservación del Águila Perdicera en Andalucía (I)*.* Technical report, Egmasa-Consejería de Medio Ambiente, Junta de Andalucía, Jaén. 657 p.
10. Ellis DH (1979) Development of behavior in the Golden Eagle. Wildlife Monogr 70: 3–94.
11. Collopy MW (1984) Parental care and feeding ecology of golden eagle nestlings. Auk 101: 753–760.
12. Collopy MW (1986) Food consumption and growth energetics of nestling golden eagles. Wilson Bull 98: 445–458.
13. Gil-Sánchez JM (2000) Efecto de la altitud y la disponibilidad de presas en la fenología de puesta del Águila-azor Perdicera (*Hieraaetus fasciatus*) en la provincia de Granada (SE de España). Ardeola 47: 1–8.
