# Supplementary material for: Laying the Foundations for a Human-Predator Conflict Solution: Assessing the Impact of Bonelli's Eagle on Rabbits and Partridges
Source: PLoS One. 2011 Jul 27;6(7):e22851. doi: 10.1371/journal.pone.0022851 (PMC3144957; doi:10.1371/journal.pone.0022851)
Supplement: Text S2 — Sensitivity analysis of the impact estimates. (DOC) [file pone.0022851.s006.doc]

**Text S2.** Sensitivity analysis of the impact estimates.

Before presenting the results, the sensitivity of each of the terms in the Lindén and Wikman (1983; [1]) equation was checked, such that the influence of the error in each parameter in the final result of the impact estimate was quantified. For this, the variation in the kill rate was calculated by applying known errors (of 1%, 5%, 10%, 20% and 50%) to each of the terms separately (see a similar approach in [2]). The data from rabbits from spring 2003 in the ISA were used as the sample. The effect of these same errors in the resulting predation rate was also evaluated. In this case the effect of variations in the prey density parameter was also quantified.

The analysis indicated that the term whose precision most affects the final result is the proportion of prey biomass in the eagle’s diet (*PPB*), such that the error passed on in the equation was almost exactly the same as the original error in this term. In contrast, the kill and predation rates were relatively unaffected by errors in the dietary consumption of females (*CF*), males (*CM*) and chicks (*CY*). The corrected prey weight (*PW*) and prey density (*DP*; obviously, this parameter only affected the predation rate) showed intermediate influence (see Table S3). However, only the errors in *DP* were considered when establishing error ranges in predation rates, given that this parameter was considered to be the most susceptible to error (see Text S3). The resulting values from errors of 10% and 50% (considered to be as “moderate” and “extreme” errors, respectively) in the prey density were finally used to offer maximum - minimum ranges in the estimates of predation rates.

**References**

1. Lindén H, Wikman M (1983) Goshawk predation on tetraonids: availability of prey and diet of the predator in breeding season. J Anim Ecol 52: 953–968.
2. Philipps RA, Thompson DR, Hamer KC (1999) [The impact of great skua predation on seabird populations at St Kilda: a bioenergetics model](javascript:submit_form()). J Appl Ecol 36: 218–232.
