# Supplementary material for: Laying the Foundations for a Human-Predator Conflict Solution: Assessing the Impact of Bonelli's Eagle on Rabbits and Partridges
Source: PLoS One. 2011 Jul 27;6(7):e22851. doi: 10.1371/journal.pone.0022851 (PMC3144957; doi:10.1371/journal.pone.0022851)
Supplement: Text S3 — Biases in the impact estimates. (DOC) [file pone.0022851.s007.doc]

**Text S3.** Biases in the impact estimates.

The consumption calculated for Bonelli’s eagle females, males and chicks was probably quite precise, given that the control of the variables (census of each of these groups, length of the consumption period and dietary requirements, the latter being age-dependent in the case of the chicks) was high. The error in the proportion of the biomass of each prey type (rabbit or partridge) in the eagle diet, the parameter potentially most influential in the final result of the kill and predation rates (see Table S3), can also be considered as quite small, given that all the territories whose diet was analysed exceeded the minimum value needed for a robust calculation (n ≥ 20 prey items; attending to simulations performed by Jovani and Tella –2006– [1]). It is also normally accepted that the contents of the pellets are a true reflection of the dietary composition of this raptor [2], given that it does not suffer from the inconveniences due to prey duplication (which does occur in other species; e.g. [3]) and from over- and underestimation respectively, of large and small prey (a typical problem in the analysis of remains; e.g. [4-6]). Additionally, the weights of the prey considered (in the case of rabbits and partridges, age class- and sex-dependent respectively) were relatively precise, especially in the case of the heaviest species (see Text S1), such that converting the relative frequencies into biomass terms was probably quite reliable. In contrast, errors in the prey census data existed, given that evidence of the presence of rabbits and partridge (middens of droppings and direct observations during or outside census walks; pers. obs.) was encountered in several transects with no counts recorded for rabbits or partridges. In the same vein, we would also expect a degree of error in the remaining transects, which would be particularly influential in those with fewest contacts. However, this source of error, more important in the case of the rabbit, was precisely that taken into account when calculating the impact (see Text S2). On the other hand, and importantly, this parameter (prey densities), which only affected the predation rate, is not the most influential in the final result of the estimate (Table S3).

Various reasons lead to our estimates of impact being conservative. Firstly, males were considered to need the same dietary requirements as the females, but due to their smaller size, it is likely that they actually require less (e.g. [7]). The spring censuses were undertaken at the mid-point of the 100 day period, and consequently the number of partridges recorded was less than that initially present, due to accumulated mortality. Regarding rabbits, although their density was probably somewhat higher at the moment of the census than at the beginning of the eagle’s breeding period, it should be remembered that this species breeds throughout the spring [8], which would largely compensate this census underestimate. Additionally, several authors have noted that the rabbit population recorded by the census is actually less than the real one, since the younger individuals are more difficult to detect [9-12] and the population visible on the surface is estimated to be 5-57% of the total population, depending on the time of year [13] –and clearly from the rabbit population (and game production) perspective, what is truly relevant is the impact on its total population, and not on that just visible on the surface–. For its part, we did not take into account in our calculations of rabbit consumption the extra-weight due to embryos in pregnant rabbit females, and pregnant females must be frequent in the studied population, at least in spring. Furthermore, the error interval introduced a priori into the predation rates (based on errors of 10% and 50% on the prey densities) was generous (see [14]).

In contrast, those individuals injured but not eaten, and which finally die from this cause are not included [15-17], nor the young, chicks or embryos which die as a result of the capture of one of their parents [18,19]. This said, the behaviour of killing appreciable quantities of prey without consuming them (‘surplus killing’), and frequent in some small carnivorous mammals [15,16], has not been described for the Bonelli’s eagle. The true sex ratio of the partridges consumed in the autumn was probably closer to a balanced ratio (1:1) than in the spring [20], and given that the males contributed more towards the biomass ingested, this may have lead to some underestimation in the total number of partridges consumed by eagles in the non-breeding period. However, the lower impact on this species in the autumn than in the spring would reduce the relative importance of this error factor.

As an overall conclusion, the final values of our estimates can be considered realistic or, more plausibly, conservative, especially in the case of rabbits.

**References**

1. Jovani R, Tella JL (2006) Parasite prevalence and sample size: misconceptions and solutions. Trends Parasitol 22: 214–218.
2. Real J (1996) Biases in diet study methods in the Bonelli´s eagle. J Wildlife Manage 60: 632–638.
3. Rosenberg KV, Cooper RJ (1990) Approaches to avian diet analysis. Studies in Avian Biology 13: 80–90.
4. Simmons RE, Avery DM, Avery G (1991) Biases in diets determined from pellets and remains: correction factors for a mammal and bird-eating raptor. J Raptor Res 25: 63–67.
5. Mersmann TJ, Buehler DA, Fraser JD, Seegar JKD (1992) Assessing bias in studies of bald eagle food habits. J Wildlife Manage 56: 73–78.
6. Sánchez-Zapata JA, Calvo JF (1998) Importance of birds and biases in diet study methods of Montagu’s Harriers *Circus pygargus* in southeast Spain. J Raptor Res 32: 254–256.
7. Nagy KA (2001) Food requirements of wild animals: predictive equations for free-living mammals, reptiles, and birds. Nutrition Abstracts and Reviews, Series B 71: 21R–31R.
8. Soriguer RC (1981) Biología y dinámica de una población de conejos (*Oryctolagus cuniculus* L.) en Andalucía occidental. Doñana, Acta Vertebrata 8 (special vol.): 1–377.
9. Parer I (1982) Dispersal of the Wild Rabbit *Oryctolagus cuniculus*, at Urana in New South Wales. Aust Wildlife Res 9: 427–441.
10. Gilbert N, Myers K, Cooke BD, Dunsmore JD, Fullagar PJ, et al. (1987) Comparative dynamics of Australasian rabbit populations. Aust Wildlife Res 14: 491–503.
11. Pech RP, Sinclair ARE, Newsome AE, Catling PC (1992) Limits to predation regulation of rabbits in Australia: Evidence from predator-removal experiments. Oecologia 89: 102–112.
12. Robson DL (1993) Natural mortality of juvenile rabbits (*Oryctolagus cuniculus*) in North Canterbury, New Zealand. Wildlife Res 20: 815–831.
13. Wallage-Dress JM (1989) A field study of seasonal changes in the circadian activity of rabbits. Z Säugetierk 54: 22–30.
14. Philipps RA, Thompson DR, Hamer KC (1999) [The impact of great skua predation on seabird populations at St Kilda: a bioenergetics model](javascript:submit_form()). J Appl Ecol 36: 218–232.
15. Oksanen T (1983) Prey caching in the hunting strategy of small mustelids. Acta Zool Fenn 174: 197–199.
16. Oksanen T, Oksanen L, Fretwell SD (1985) Surplus killing in the hunting strategy of small predators. Am Nat 126: 328–346.
17. Mittler J (1997) What happens when predators do not completely consume their prey? Theor Popul Biol 51: 238–251.
18. Mañosa S (1991) Biología tròfica, ús de l´hàbitat i biología de la reproducció de l´astor *Accipiter gentilis* (Linnaeus, 1758) a la Segarra [PhD thesis]. Barcelona: University of Barcelona.
19. Gilg O (2002) The summer decline of the collared lemming, *Dycrostonyx groenlandicus*, in high arctic Greenland. Oikos 99: 499–510.
20. Moleón M, Gil-Sánchez JM, Real J, Sánchez-Zapata JA, Bautista J et al. (2007). Non-breeding feeding ecology of territorial Bonelli’s eagles *Hieraaetus fasciatus* in the Iberian Peninsula. Ardeola 54: 135–143.
